# Opportunities in low-level radiocarbon microtracing: applications and new technology

**DOI:** 10.4155/fso.15.74

**Published:** 2015-12-23

**Authors:** Le Thuy Vuong, Qi Song, Hee Joo Lee, Ad F Roffel, Seok-Ho Shin, Young G Shin, Stephen R Dueker

**Affiliations:** 1 Sivvon Biolabs, 1520 E, Covell 311, Davis, CA 95618, USA; 2 BioCore Co., Ltd, Seoul, IT Mi-Rae Tower, 8F (33 Digitalro 9-ghil) #60-21 Gasan-dong, Geumcheon-gu, Seoul, South Korea 08511; 3 PRA Health Sciences, Stationsweg 163 9471 GP Zuidlaren, The Netherlands; 4 College of Pharmacy, Chungnam National University College of Pharmacy, 99 Daehak-ro, Yuseong-gu, Daejeon, South Korea 305-764

**Keywords:** accelerator mass spectrometry, AMS, cavity ring-down spectroscopy, microdosing, microtracing, pediatrics, radiocarbon

## Abstract

^14^C-radiolabeled (radiocarbon) drug studies are central to defining the disposition of therapeutics in clinical development. Concerns over radiation, however, have dissuaded investigators from conducting these studies as often as their utility may merit. Accelerator mass spectrometry (AMS), originally designed for carbon dating and geochronology, has changed the outlook for in-human radiolabeled testing. The high sensitivity of AMS affords human clinical testing with vastly reduced radiative (microtracing) and chemical exposures (microdosing). Early iterations of AMS were unsuitable for routine biomedical use due to the instruments’ large size and associated per sample costs. The situation is changing with advances in the core and peripheral instrumentation. We review the important milestones in applied AMS research and recent advances in the core technology platform. We also look ahead to an entirely new class of ^14^C detection systems that use lasers to measure carbon dioxide in small gas cells.

## Abstract (lay abstract)

**Lay Abstract:** Radiocarbon (^14^C) is produced continuously in the upper atmosphere and incorporated into biological systems through photosynthesis. Incorporation of ^14^C ceases at death, hence by measuring natural levels of radiocarbon in deceased organic materials the age of the objects (e.g. vegetation, wood and historical artifact, among others) can be determined. Early carbon dating determinations (starting in the late 1940s) relied on monitoring the small levels of radioactivity decay energy emerging from the sample. In the late 1970s nuclear physicists in collaboration with archeologists developed a far more sensitive and efficient measuring technique known as accelerator mass spectrometry (AMS), which can identify individual carbon atoms. The technology was extended to biomedical research in the early 1990s. Here it had an immediate impact on *in vivo* human research across diverse areas such as carcinogenesis and human nutrition. The same carbon dating technology now supports human ^14^C-tracer metabolism studies conducted as part of early drug development. The high sensitivity of AMS enables scientists to explore new drug candidates safely in any human subpopulation at very low chemical (microdosing) and radioactive doses (microtracing). A ‘big physics’ tool originally developed for dating archeological artifacts is now benefiting modern drug development in unanticipated ways. The full benefits of the sensitive measurement technology were impeded by the scale and operational complexity of the early instruments, but this situation has changed significantly over the last decade after the emergence of purpose-built biomedical instruments. The evolving relationship between radiocarbon, AMS, and modern drug development is discussed in this review.

**Figure 1.**
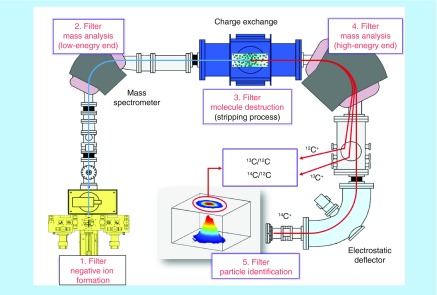
**A schematic of the 200 keV ‘MICADAS’ accelerator mass spectrometry developed at ETH Zürich (footprint: 2.5 × 3 m).** Core concepts of ^14^C-accelerator mass spectrometry are summarized: negative ion production to eliminate the atomic isobar, low-energy mass filtering, high-energy collisions to eliminate molecular isobars, high-energy dipole mass spectrometry resolution of carbon isotopes to 1 amu, particle identification of ^14^C after electrostatic sector filtering. Ion beams of ^12^C and ^13^C are measured in off-axes Faraday cups.

**Figure 2.**
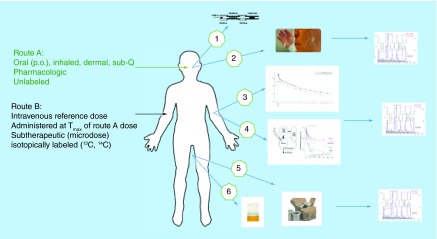
**Information-rich absolute bioavailability using an intravenous ^14^C-microdose.** The primary objective of conducting a microdose absolute bioavailability study is to obtain information on the absolute bioavailability and compound clearance. Other valuable information is available that can help complete the drug disposition picture. The total radioactivity in expired air, bile, plasma or whole blood, cell populations or small biopsies, feces and urine are all available for specific analyses without extensive, matrix-specific assay development. Small quantities of diluted or processed matrix can be profiled on chromatographic systems for metabolite profiling from any matrix. sub-Q: Subcutaneous.

**Figure 3.**
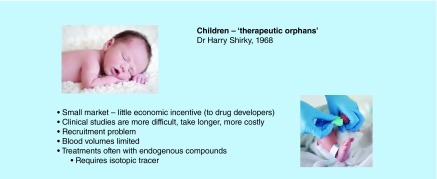
**Therapeutic orphans.** The pediatric community remains from several years to decades behind the adult population in terms of drug development. As a result, children are disproportionately treated with older, less modern drugs. Over 40 years ago, Shirkey recognized the dilemma of pediatric drug labeling, called therapeutic orphans, to capture its concept. There are many hurdles to overcome in conducting a pediatric versus an adult study. Available blood volumes for testing in particular are limiting. Accelerator mass spectrometry sensitivity allows accurate and precise determination of drug product in small microliter blood volumes [57].

**Figure 4.**
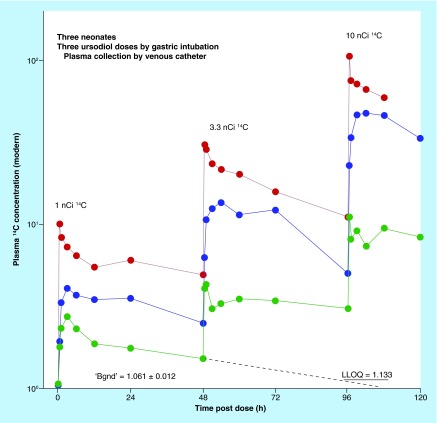
**Biokinetics of ^14^C-ursodiol microdoses in neonates.** The figure plots the ^14^C concentration in 25 μl samples of plasma from three neonates, each dosed at 1, 3.3 and 10 nanoCi of ^14^C-ursodiol with 48 h between doses. Background natural ^14^C was measured in an initial cohort of five subjects. The LLOQ was determined at six-times the standard deviation in the background measurement. This proof-of-concept study suggested that accelerator mass spectrometry techniques are applicable to the study of any medications prescribed to newborns.

**Figure 5.**
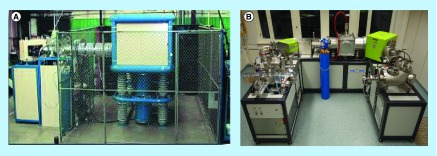
**Compact, low-maintenance accelerator mass spectrometry systems.** **(A)** The single stage open stack accelerator mass spectrometry (AMS) system unit from National Electrostatics Corporation is an air-insulated single stage accelerator that is simple in design. The instrument is robust and easy to maintain as it does not have any moving parts or pressurized insulating tank around the high-voltage section. The combination of simple design, excellent performance and reasonable price has resulted in 12 new National Electrostatics Corporation AMS installations as of 2013. **(B)** With dimensions of only 3.4 m × 2.6 m × 2 m, the pictured green MICADAS analyzing dating AMS system is the most compact commercially available ^14^C-AMS system. Fixed magnets are used in place of electromagnets, reducing the overall footprint and power consumption. In conjunction with the gas interface system, this AMS system performs fully automated gas measurements with an autosampler, an elemental analyzer or CO_2_ filled glass or quartz tubes (provided by ETH).

**Figure 6.**
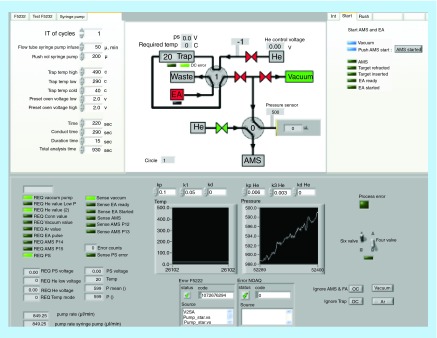
**Control module for the automated CO_2_ analyzing system.** The system omits sample preparation of total ^14^C determinations with self-directed analysis for up to 200 samples which is represented in the middle top panel. The hybrid ion source accepts both solid and gaseous samples. Small samples (<2 µl of plasma) are placed in tin foil cups, and the cups are placed in a carousel. The analyzer combusts the samples one by one and the generated oxidative gases are guided by the carrier gas (helium) to a trapping matrix (zeolite). The zeolite is desorbed using heating and passed to a syringe that precisely feeds the gas into the accelerator mass spectrometry ion source after mixing with helium gas to a precise ratio (courtesy of W Vaes).

**Figure 7.**
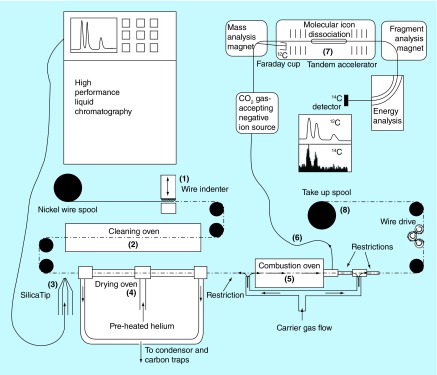
**Schematic layout of liquid chromatographic/accelerator mass spectrometry.** A wire indenter generates periodic indentations on the wire. Surface carbon is removed and the wire is oxidized at high temperature. A stream of effluent or a single droplet is placed on the wire. Solvent is evaporated at elevated temperature in an atmosphere of helium. Nonvolatile analyte is combusted at high temperature in a helium and oxygen atmosphere. The flow of gas through the combustion oven directs all combustion products to the gas-accepting ion source through a narrow i.d. fused silica capillary. Reused with permission from [78], © American Chemical Society (2015).

**Figure 8.**
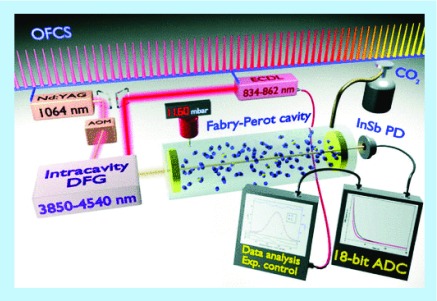
**Diagram of experimental setup of cavity ring-down system.** The basic components of a cavity ring-down spectroscopy system are a tunable laser source (infrared light region), a high-finesse optical cavity that bounces the light between highly reflective mirrors and a data acquisition system. ^14^C^16^O_2_ optical detection is based on molecular absorption spectroscopy saturated-absorption cavity ring-down (discussed above). An optical cavity filled with CO_2_ is illuminated with an intense laser tuned to excite the targeted rotovibrational transition of ^14^C^16^O_2_. When the laser is turned off, the radiant energy stored in the cavity ‘rings down’ or decreases over time. Within this ring down rate the absolute quantity of ^14^C^16^O_2_ is discerned. The prototype system has a footprint of 2 m^2^ and is expected to have a cost of less than a mass spectrometer. The currently described system requires large sample masses (68 mg carbon) and long acquisition times (hs) to achieve results similar to accelerator mass spectrometry. With further improvements, the infrared technique may well become the method of choice for measuring the isotope ratios of many common elements. ADC: ntibody–drug conjugate. Reproduced with permission from [81], © The American Physical Society.

## Introduction to radiocarbon, AMS & pharmaceutical ADME

The pharmaceutical industry has been challenged through regulatory initiatives to find ways for increasing productivity while improving safety. The ability to generate metabolism and relevant dispositional information prior to or within early clinical development has the dual benefit of reducing animal testing and improving clinical success rates by reducing failures stemming from unfavorable dispositional properties (improvement in drug-like qualities). Even a marginal improvement in clinical success rates could have a substantial benefit in terms of the availability of new drugs [1].

After its discovery in 1940, radiocarbon ^14^C quickly became the most useful isotopic tracer for organic compounds in biochemistry [2]. This long-lived (5730-year half-life) radioisotope offered maximum potential for stable molecular inclusion with an inherent means of quantitation through the counting of energetic electrons emerging from the sample. For pharmaceutical and biomedical applications, ^14^C labeling enabled the universal application of isotopic quantitation without extensive or specific method development, with the assurance that all derivatives of properly labeled compounds (metabolites) could be quantified equivalently without reference standard materials for individual components [3,4].

Radioactive decay counting is a natural method of choice for the detection of radionuclides. Long-lived radionuclides, however, decay slowly, and the data acquisition time required to quantify their activity at ambient levels is impractically long for routine bioanalysis. To compensate for inefficient detection, human radiolabeled drug studies uses doses that are large multiples of the ^14^C naturally present in the human body (a 70 kg adult contains about 100 nanoCuries of ^14^C). Indeed, human metabolism studies using ^14^C-labeled compounds are normally performed with 100–200 µCi of radioactivity, a level 1000–2000-times the total natural burden of radioactivity attributed to ^14^C in an average-sized adult [3].

Accelerator mass spectrometry (AMS) efficiently measures ^14^C and select other rare radioisotopes using atomic mass spectrometry. AMS is a form of isotope ratio mass spectrometry that filters and separates ions of atomic carbon using magnetic and electric fields at high kinetic potentials (100–10,000 kV) [4,5]. The sensitivity of the technique depends on engineered quantities such as ionization efficiency and ion optics that are independent of the physical constraints of isotope half-life [4]. Limits of detection are expressed in terms of attomole (10^-18^) to zeptomole (10^-21^) quantities of ^14^C in small samples, which translate to a four to five order of magnitude increase in sensitivity over ^14^C radioactive decay counting. Accordingly, human clinical tests can be performed at radiochemical exposures that do not differ significantly from the natural background of radioactivity. In Figure 1 we present a diagrammatic representation using one spectrometer’s configuration (MICADAS) that highlights the fundamental sector components of an AMS instrument and the data output (carbon isotope ratios) [6].

Early biological AMS investigations in the early 1990s were used to estimate the turnover times of elastin in cadaver lung tissues [7] and genotoxic reactive metabolites of environmental origin that were undetectable in circulation [8,9]. Since then, AMS has been used to investigate low-dose exposure to agricultural chemicals [10], identify the biomolecular targets of ^14^C-organophosphate toxins [11], quantify tissue turnover rates [12–14], determine the metabolic fate of vitamins and essential dietary chemicals in women of child-bearing age [15–17] and assess the biliary status of week-old neonates using labeled bile acids [18,19].

For pharmaceutical development, radiolabeled compounds are applied to understand the absorption, distribution (limited application in humans), metabolism and elimination (ADME) characteristics of a development compound in laboratory animals and later humans. The radiolabel provides a means for quantitation without compound-specific method development, assay validation or even reference materials. The standard pathway for a human clinical test is to dose 100–200 µCi (10^-6^ Ci) of radioactivity in a small cohort of volunteers (often middle-aged males are preferred), usually after Phase IIA efficacy trials, with complete collection of excreta (urine, feces, sometimes expired air) for calculating the mass balance (recovery) and of the administered compound [20,21]. Whole blood and plasma are analyzed for parent or active drug concentrations, total radioactivity (metabolite burden) and metabolites (metabolite profiling). A small percentage of these studies, for reasons which are presented in a later section (see section 'New applications: mass balance at microtrace ^14^C levels'), are now conducted by necessity or design with radioactive dose levels that are too low to be supported by decay counting. Often these radioactive doses fall into a range low enough to exempt them from radioactivity burden calculations (animal and human dosimetry calculations) and certain regulatory oversights. The term ‘microtracer’ or ‘light-label’ has been aptly used to differentiate this safer design from the more conventional approach [22–24].

A recent clinical innovation often dependent on ^14^C-radiolabeled compounds is the concept of sub-therapeutic dosing or microdosing. A *‘*‘microdose*'* is a regulatory defined term (codified in the ICH M3(R2) guidance [25]) that references a clinical drug dose that is 1/100^th^ of the expected pharmacologically active dose and ≤100 µg. Microdose studies conducted before the conventional safety and efficacy testing, or so-called Phase 0 studies, were intended to assist in the selection/deselection and optimization of drug candidates in humans in place of extensive and sometimes misleading animal-based evaluations. Microdosing was closely associated with AMS from the start as the successful implementation of microdosing required the high sensitivity (femtomolar range) available with AMS quantitation. Today, microdose investigations are sometimes supported by state-of-the-art LC/MS technology and stable-labeled microdoses. These studies are generally more limited in scope relative to AMS-based microdose tests [26].

Microdoses are applied for two common study types: for phase 0 candidate selection and for microdose absolute bioavailability studies. In the latter, an intravenous (iv.) microdose is administered concomitantly with a pharmacologic dose given by the intended route to assess absolute bioavailability. Several terms are used to identify this type of absolute bioavailability study: microdose absolute bioavailability, iv. microtracer (this term may be trademarked to Quotient Clinical but is often used by others) and simultaneous iv. micro + oraltherap studies [22]. These regulatory studies are becoming a new norm for the industry while the pure phase 0 concept is not widely used.

In our view, the pharmaceutical industry remains to leverage the full potential of AMS. While early concerns over the analytical performance of AMS and the acceptance of the AMS data by health authorities have largely dissipated, lingering objections over analytical costs and sample turnaround times persist. These objections may be partially or wholly allayed by the availability of simpler more automated systems, some of which are presented later in this manuscript. There are incentives for developers to innovate the AMS or radiocarbon detection platform in general. Some incentives include: new regulatory positions that emphasize pharmacokinetics and early metabolism assessments [25,27], ever more complex bioanalytical challenges as drugs grow in potency and molecular diversity (e.g., biologics); and high demand for sensitive ^14^C detection from the earth sciences (e.g., carbon cycle research), radiocarbon dating and carbon emissions monitoring [28]. The field of sensitive ^14^C detection is truly broad in scope, given that carbon lies at the center of all biological systems.

The direct aims of this manuscript are to highlight key clinical applications that are driving technological innovation in sensitive ^14^C tracing and typify the unique data that sensitive quantification radiocarbon tracers enables for pharmaceutical development. We highlight areas that are uniquely addressed by AMS, including microdose absolute bioavailability, the mass balance of difficult molecules, pediatric pharmacokinetics and metabolism, and the broad class of biologic molecules. We also discuss some of the favorable advances on the AMS technology platform and the latest developments in the optical laser systems that may eventually offer an alternative to AMS for radiocarbon detection. This review should be viewed as a partial rather than a comprehensive, as the types and numbers of AMS applications are diverse in scope [24,29–30].

## Applications: absolute bioavailability: opportunity from a requirement

Often, discussions of AMS will begin with human microdosing. Microdosing (when used for kinetic information and not molecular imaging), emerged in the early 2000s as an opportunity to understand the human pharmacokinetics (and metabolism) of a molecule or class of molecules prior to investment in a complete Phase 1 development program. Early and safe human screening prior to standard Phase 1 testing would presumably reduce the reliance on animal models or human cellular assays that lack the full metabolism and target complement of a living person for candidate selection and optimization. The concept appeared sound. The utility of the technique however was dependent on the ability of a sub-therapeutic microdose to well predict the later pharmacokinetics of the therapeutic dose [31,32]. Clinical validation studies recently concluded in Europe have found oral microdosing to be ∼70% predictive for over 20 molecules tested and the data for iv. microdosing are even better [33–35].

The link between microdosing and AMS technology was initially one of assay sensitivity: AMS was viewed as the detector capable of routinely measuring the femtomolar concentrations of drug analyte in tissues and fluids following a human microdose. Useful pharmacokinetic data however required that any analyte be cleanly separated from other drug related components in the sample prior to determination of the ^14^C contents, an activity not required for carbon dating. The total assay accuracy (and precision) was then dependent on the quality of relatively 'low tech' separation procedures that could not be internally standardized. This led to an unusual relationship between a bulk detector of ^14^C for carbon dating and single molecule drug pharmacokinetics. To be useful for pharmacokinetics, a series of compound isolation steps had to be taken (e.g., extraction, solvent concentration, off-line liquid chromatography with fraction collection, etc.) before the separate AMS sample definition procedure (e.g., graphite formation). The engineered high accuracy and precision of AMS which made it suitable for carbon dating was thus reduced to the precision of the least reliable steps (overwhelmingly, the basic wet laboratory procedures). AMS was a powerful, but unlikely (cumbersome) tool for quickly exploring pharmacokinetics in a discretionary Phase 0 study.

AMS may have remained at the fringes of clinical development had it not become integral to addressing one specific regulatory expectation, the Absolute Bioavailability test. Regulatory requirements can have a greater and more immediate impact on technology development than advances in basic science (as nicely stated by Salehpour [36]) and this is certainly the case for AMS). Whereas Phase 0 microdosing is an internal choice for the industry, regulatory pressure for absolute bioavailability data as part of a New Drug Registration prompted the industry to engage AMS technology.

“*Regulatory requirements may have a bigger impact on the evolution of this technology in clinical science than any technical or scientific improvement in the area of tracer syntheses or analytical chemistry*.” [36].

An instructive example of the positive effects of regulatory pressure appears in the editorial by Arnold and LaCreta [37], ‘When opportunity met aspirational goals: accelerator MS, microdosing and absolute bioavailability studies’. This article presents an industry perspective on how one industry group learned to leverage ‘an esoteric carbon dating tool…’ with favorable regulatory guidance, after the Australian Therapeutic Goods Administration restated its position that information on absolute bioavailability would be ‘required’ for all new chemical entities.

Absolute bioavailability studies are performed to provide an understanding of the systemic availability of a non-intravenously administered drug versus an iv. reference dose that is 100% available. The industry, according to Arnold and LaCreta, became quite skilled at finding workarounds to these studies for reasons owing to the time and cost with developing a separate pharmacologic iv. formulation and supporting iv. toxicology data – a costly, time-consuming project for a molecule not destined to be administered by that route outside of this single test.

The AMS method offers a very simplified approach in terms of iv. dose manufacture, abbreviated toxicology requirements and conduct of the clinical study in a single cohort without a crossover design. An iv. microdose is administered concomitantly with the oral therapeutics dose in a single test period. The *in vivo* mixing of the microdose with the absorbed cold compound ensures relevant therapeutic kinetics of the microdose. Seeing AMS as a potentially core analytical technique in support of these studies, the industry group commissioned what is arguably one of the more detailed evaluations of the performance of the AMS technique. Details on the development of the method, technique-appropriate validation and eventual bioanalysis of clinical study samples can be found in the companion paper in the same issue titled ‘Overcoming bioanalytical challenges in an Onglyza™ iv. (^14^C) microdose absolute bioavailability study with accelerator MS’ [26].

This study and the associated articles present an interesting account of how a regulatory challenge in the form of a requirement transmogrified into an opportunity to consider ‘faster, better and/or cheaper alternatives to the more comfortable traditional approaches’. Microdose absolute bioavailability serves as a frequent entry point to AMS for companies without prior AMS experience [38]. This is an example of regulatory requirement leading to an innovation that should deliver broad and lasting effects.

“*Leveraging the regulatory advantages of microdose administration can save many months, resources and millions of dollars in costs – clearly an advantage from both a time and cost perspective*.” [37].

### Assay validation, qualification, technique-appropriate validation?

Interest in microdose absolute bioavailability cast AMS into the area of regulated bioanalysis and required some of main commercial contract research organizations (CRO) providing AMS analysis (such as Accium, Xceleron, Vitalea Science) to retool (personal experience) their bioanalytical procedures and implement practices found in validation assays for LC/MS and ligand-binding. The Onglyza™ and other parallel work [39] clearly established that the AMS methodology can meet or exceed performance criteria required for LC/MS in regulated bioanalysis. The level of pre-study assay validation found in that study had not been a standard practice at that time. Today, more-or-less similar practices are being conducted by leading providers of AMS services and several industry groups, such as the Global Bioanalysis Consortium [40] and European Bioanalysis Forum [41], are currently collaborating to standardize views on performance expectations for the AMS methodology and what activities constitute a ‘validated’ assay for AMS-based pharmacokinetic studies [42–46]. What has been clear from our participation in these and other discussions is that the performance quality of a method lies almost exclusively with basic extraction and isolation procedures and has little to do with the AMS instrument performance. For example, standard plots or ‘calibration curves’ only adjust for recovery losses during extraction and are not used to adjust for differences in the instrument AMS response due to matrix and tuning effects. Normalization for recovery losses can more efficiently be compensated for through the use of single-level recovery standards rather than multilevel plots that do little to improve the accuracy of the dataset (personal opinion of some of the authors of this paper). It is disadvantageous and inappropriate to the field of AMS to apply the same approaches required for proportional measurement techniques such as LC/MS. Since AMS shares many of the same characteristics as liquid scintillation counting (LSC), practices applied here may be more appropriate. It will be interesting to watch how these harmonization activities play out and if a single standardized approach to assay validation, qualification or technique-appropriate validation, whatever the final term used, is reached.

### Information-rich absolute bioavailability in a single test

There lies a wealth of dispositional information beyond the core clearance and bioavailability parameters available from an iv. radiolabel study assuming a sensitive detection method. Inclusion of secondary absorption, distribution, metabolism and excretion or ADME endpoints within an absolute bioavailability has been casually referred to as being information-rich [47]. Available information includes: total radioactivity (TRA) in plasma for assessment of metabolite burden and potential for strong or covalent binding, urinary TRA as a measure of excretion, TRA in target and in off-target tissues and metabolite profiling (see Figure 2). Any matrix in principle is accessible to examination assuming specimen collection is written into the clinical protocol. A review of the activities at GlaxoSmithKline highlights various secondary ADME objectives that have been tagged onto both microdose absolute bioavailability and conventional pharmacokinetic studies [24,47]. Quantifying circulating and excretory metabolism after an iv. administration is advantageous as it provides a purer assessment of the postabsorptive pathways and metabolic clearance under conditions not confounded by gut wall and hepatic first pass metabolism.

One new ADME endpoint in a microdose absolute bioavailability study stands out for mention. This is the option to assess biliary excretion after an iv. microdose using a minimally invasive sampling tool referred to by its commercial name, the Entero-Test™. The Entero-Test™ is a marketed device originally developed for sampling to support clinical diagnostic assays [47]. The peroral string assay is a simply designed method performed by collecting bile acids on a suspended nylon string taped to the corner of the mouth. Following a period of about 5 h postswallowing, the string and gastrointestinal fluid absorbed within it are withdrawn through the mouth. The biliary fluid is then solvent desorbed from the string and analyzed for bulk ^14^C and for metabolites by radiochromatographic profiling. The absence of detectable ^14^C on the string would eliminate further consideration of biliary excretion and help validate the relevancy of the chosen safety species. Metabolite profiles would be uncomplicated by bacterial metabolism, assisting in the prediction of the potential risks of drug–drug interactions with co-administered medication. This information typifies the value that can be derived in microdose absolute bioavailability studies with minimal incremental costs.

## New applications: mass balance at microtrace ^14^C levels

Human mass balance studies that use radiolabeled drug products are a standard part of the development process for new drugs [42]. These investigations are infrequent, however, in part due to the time and cost of obtaining approvals for high radiation dosing to humans. While these costs may be small in terms of total costs associated with drug development, the odds of clinical failure and the need to adhere to strict clinical timelines can discourage investment in radiolabeled tools. However, radiotracer studies are more definitive and the data are easier to interpret than in nonlabeled studies. Many development teams would appreciate early ADME information if there were few barriers. AMS sensitivity has expanded the scope of conditions where a radiolabeled molecule can be safely applied and often with fewer barriers. For ease of discussion, we segregate this expansion in microtracer scope into three categorical areas:

Category 1: for enhanced assay sensitivity. In particular when dosimetry or assay sensitivity is limiting;
Category 2: for expedited clinical time frames. When there is insufficient time to fulfill preparative requirements for traditional radiolabeled studies. Human dosimetry and GMP radiolabel are not hard-and-fast requirements;
Category 3: for expanded patient/volunteer populations. Previously underserved subpopulations, for example, children, women of child-bearing potential, pregnant women and infirmed patient populations.

Category 1 is relatively clear. There are limits of the amount of radioactivity that can be administered to human subjects due to the negative effects of ionizing radiation when absorbed by tissues (dosimetry). In particular for drugs that are of high volume of distribution and/or extensively metabolized, slowly eliminated, or that tend to concentrate in specific tissues (e.g. melanin binding in the eye), the magnitude of the permissible radioactive dose can be insufficient to overcome assay sensitivity limitations set by decay counting. One such example is the long half-life drug, such as vismodegib. Vismodegib is the first Hedgehog signaling pathway-targeting agent to gain US FDA approval. It is a small molecule but has a unique pharmacokinetic profile and a long elimination half-life of 12 days [43,44]. Multiple factors were considered to contribute to its unique pharmacokinetics properties, such as nonlinear absorption, saturable high-affinity binding to alpha-1-acid glycoprotein and extremely low clearance. A conventional mass balance study could not be ethically performed over radiative exposure considerations. Moreover, very low circulating concentrations would challenge assay sensitivity limits imposed by decay counting. Therefore, a microtracer mass balance study using AMS was performed in healthy volunteers, allowing quantification of radioactivity in plasma and excreta over a 56-day study duration. The mass balance study with oral administration of 150 mg vismodegib admixed with 1 µCi of ^14^C-Vismodegib indicated that on average 82% of the dose was recovered in feces with 4% recovered in urine. This was believed to be excellent recovery for a compound with such a long half-life.

Microtracer investigations have fewer requirements than traditional radiolabeled studies prior to initiation. Category 2 applies to instances where a radiolabeled study must be initiated within expedited time frames or when human dosimetry is not normally determined. We have observed several conditions that can necessitate expedited timeframes: when there is a decision to add a microtracer dose in a Phase 1 dose escalation study and supporting tests to support human dosimetry calculations have yet to be initiated [29,45], in response to an agency request when existing drugs are being repurposed (personal experience of some of the authors) and for human nutritional or food products that normally do not undergo a pharmaceutical-styled development program.

We have observed two favorable trends in low ^14^C radiolabeled studies that control cost within accelerated timelines. First, is the practice (RDRC in the USA, ARSAC in the UK and an approved practice in the Netherlands) to exempt microtrace radioactive doses from a requirement for supporting human dosimetry (∼1 µCi or 0.037 MBq though higher radioactive doses have also been exempted on a case-by-case basis; personal communications with CROs in USA, UK and the Netherlands). Second, is the option for using microtrace levels of radiolabeled compound that are high quality but do not carry a GMP certificate (reduced expense). One argument for non-GMP radiolabel is that the mass of the radiolabel is at impurity levels relative to the unlabeled active pharmaceutical ingredient (API) and microdose levels of manufacturing control should apply [46].

Relevant guidance can be found in Europe under EudraLex Volume 4 [48] and in the FDA under its Guidance for Industry cGMP for Phase 1 Investigational Drugs [49]. These documents provide some flexibility in the controls applied to the manufacture of Investigational Medical Products or Drug Products for use in early clinical trials, stating that controls should be consistent with the stage of development of the drug product. The emphasis is placed on well documented and scientifically sound approaches for ensuring drug purity and consistency in manufacturing. Small quantities of radiolabels are proportionately easier to purify and characterize for impurities using a combination of radiochromatographic HPLC and supporting mass spectrometry and/or nuclear magnetic resonance, than cold (unlabeled) drug product. Clinical CROs will often initiate their own internal guidelines given the flexibility afforded by the guidance documents. For example, one clinical CRO (AF Roffel, PRA Health Sciences) has the following internal guidance policy regarding under what conditions a well-characterized but non-GMP API would be acceptable: ‘In all microdose studies and in full-dose human ADME studies, assuming that the ^14^C-API is less than 0.15% of the total dose of the API’. Other CROs will define their limits differently, and the trial sponsor assumes ultimate responsibility for the quality of investigational medicinal products. The key point is that there is flexibility in the manufacturing of the radiolabeled product and it is paramount to have scientifically sound and defensible justification for choosing any approach, particularly if it falls outside of standard GMP or GMP-like practices. Many AMS studies in our collective experience have utilized non-GMP radiolabel; we are not aware of circumstances where the level of documentation associated with the synthesis negatively impacted the study.

Category 3 addresses what are sometimes referred to as underserved subpopulations. These include children, women of child-bearing years and even patient populations normally excluded from radiolabeled investigations. Experiments with AMS administer ^14^C doses as low as a few picoCuries per gram to informed volunteers. The level of radiation thus imposed is much lower than the commonly accepted exposure incurred during air flight, etc. Human nutrition and now neonatal investigations clearly show that any subpopulation is available for testing at microtrace ^14^C levels with minimal risk [18].

## Following ALARA

The FDA and the Nuclear Regulatory Commission jointly regulate human ADME testing in USA. As regulations provided the motivation for bringing companies to consider the iv. microtracer approach for absolute bioavailability, the same could hold true for the low radiolabeled human testing in general. The USA code of Federal Regulations states the principle of As Low As Reasonably Achievable (ALARA) for the dosing of human subjects [50].

“*The amount of radioactive material to be administered shall be such that the subject receives the smallest radioactive dose with which it is practical to perform the study without jeopardizing the benefits to be obtained from the study*.” (21CFR361.1).

Stricter adherence to the principles of ALARA would effectively establish microtracing as a universal low ^14^C platform for human studies. Such a position is only reasonably conceivable after trace radiocarbon detection methods are as simple and convenient to implement as modern LSC or LC/MS methods. A substantial shift to microtracer levels would have the dual benefit of bringing quantitative tracer testing to all subpopulations while providing economic incentives for technology innovation. Thus, in keeping with one theme of this manuscript regarding the impact of regulatory requirements, stricter adherence to ALARA would create short-term problems for some groups, but the advances in technology this action would catalyze broad and lasting benefits for biomedical science.

### Novel mass balance study designs

Microtrace mass balance studies serve a valuable role for select bioanalytical problems. There are also some less obvious applications that deserve mention. An excellent example is the steady-state radiolabeled mass balance study. Multiple guidance documents have expressed the importance of assessing the exposure to circulating metabolites at steady-state, a condition that appropriately applies to drugs taken chronically over long durations. In a steady-state ^14^C-AMS study, the radiolabeled dose is divided into multiple small fractions that are given over consecutive days until steady-state is obtained. This study design has been discussed for years, since AMS became available, without many industry adopters. ‘A novel study using accelerated mass spectrometry to evaluate the pharmacokinetics of total ^14^C-AL-8309 (Tandospirone) following topical ocular administration in healthy male subjects’ was the first reported use of AMS with a topically applied ophthalmic product [51]. To our knowledge, it was also the first (and perhaps only) instance where multiple radiolabeled doses were administered to steady-state conditions. The steady-state dosing protocol was chosen due to concern over accumulation of long-lived metabolites. The primary objective of this study was to characterize the excretion and pharmacokinetics of total ^14^C and metabolite concentrations following bilateral, topical ocular drops of ^14^C -AL-8309B (labeled either at the pyrimidyl ring [cohort A] position or at the imido-carbonyl ring [cohort B] position). Drops of either molecule were administered twice daily from day 1 through day 6 and once in the morning of day 7, in 16 healthy male subjects (8 per molecule cohort). Each drop (∼24 µl) of ^14^C -AL-8309B 1.75% ophthalmic solution (equivalent to 420 µg AL-8309) contained 500 nCi of ^14^C-AL-8309. The results showed moderate accumulation (1.48- to 1.86-fold) in the mean systemic total ^14^C plasma concentrations at steady-state (day 7) compared with single dose (day 1). The mean total ^14^C eliminated was 3.5-fold and 3.7-fold greater in the urine than in the feces for Cohort A and Cohort B, indicating that ^14^C -AL-8309 is primarily excreted through renal elimination. Complete details of the parent plasma concentration and metabolite profile remain to be released. This study was a first of its kind on many levels (radiolabel administered to a sensitive organ, dosing to steady-state, ocular delivery, differentially labeled molecules to account for all possible metabolites) and serves as a powerful example of the possibilities afforded by sensitive radiocarbon detection.

We note with interest that Svensson published a forward-looking article, ‘Could traditional mass balance studies in the ‘twilight zone’ be improved by means of accelerator MS measurements?’ in the same year [52]. This article not only proposed steady-state radiolabeled repeat dosing, but also a rethinking of some of the objectives of radiolabeled mass balance studies in general. One particular point is the commonly unmet goal of achieving >90% recovery (balance) in excreta (42% of 171 mass balance studies reviewed by Roffey *et al.* [42] had recoveries of less than 90% and 15% had recoveries of less than 80%).

Incomplete or low recovery is often attributed to procedural error in the collection. While this is certainly true in some cases, if 10% or greater percentages are truly accumulating in tissue reservoirs, a better objective, per Svensson, would be to identify and quantify the areas of deposition. Determination of the ‘residual fraction’ of the administered dose in the body through assessments of trace radiolabeled material in breath, urine, feces or even tissue biopsies (skin, red blood cells, lymph, muscle, fat, etc.) well after dosing would eliminate ambiguity and provide actionable information. These measurements are well within the capabilities of AMS and such measurements have been previously shown in research and pharmaceutical for research applications. AMS sensitivity enables innovative study designs that can have more far-reaching consequences than simply a reduction in radioactivity.

### Microtracers of metabolism in Phase I

Metabolite discovery and quantitation is a strength of applied AMS sensitivity and a necessary part of mass balance studies [3]. Multiple regulatory guidance documents have suggested performing human metabolite work earlier in development programs to understand the relationship between metabolites and the pharmacology and toxicity of the drug [25,53]. While companies generally have a good understanding of drugs or metabolites from animal or *in silico* work prior to human testing, neither system is complete. The late-stage discovery of unique or disproportionate human metabolites can introduce delays in the program, sometimes with major economic consequence. These concerns have prompted some companies to move their human ADME programs to earlier stages of the development program. While the overhead and preparative work for a definitive ADME study during Phase 1 dose-escalation trials might be daunting, adding a microtracer amount of ^14^C-drug to one arm of these studies for metabolite discovery and limited routes of excretion data are a path with far fewer barriers [45,54]. If one later stage delay were prevented, the practice would more than pay for itself. Moreover, early detailed knowledge of the human metabolism picture will help validate the choice made for the safety species, lead to initiation of the synthesis of major metabolites as LC/MS reference material and may even uncover the next drug product. We and others have seen more and more companies discussing, and some using, this option [29,54].

## New applications: pediatrics

Pharmaceuticals are seldom developed specifically for pediatric populations and the off-label use of existing medications is an ever-present and growing concern among the medical community. Children are not ‘mass-scaled little adults’, there are many instances where the reported difference in metabolism due to developmental changes between infants, children and adults is significant. For example: the hydroxylation reactions of diazepam are virtually lacking in both premature and full-term neonate, but important in a child [55]. Differences in drug metabolism linked to developmental changes are not merely of academic interest: many drugs are bioactivated to toxic metabolites and the differential maturation of various pathways may put the infant at risk during a limited developmental stage [56]. The lack of information on drug dosages given to infants and children has created a class of ‘therapeutic orphans’ (see Figure 3) [57]. Knowing the right dosage of a drug for a population is as important as choosing the right drug for that population [58,59].

“*The question remains: is it unethical to not properly evaluate a treatment before its use in patients, especially those whose physiology is distinctively different from the initial adult test population?*” [18].

Microtracing can have a significant impact on the currently underserved pediatric investigations where off-label dosing is common. In a microdosing study using AMS, the administered dose is typically in the nanogram range, some two or more orders of magnitude less than the therapeutic range. With the low sample volume requirements and dietary levels of ^14^C in enriched molecules, serial pharmacokinetic blood draws and balance measurements are feasible. Vuong and Blood, with coworkers, forged what may be the first pediatric (neonates) radiocarbon study in over 20 years globally (as assessed by a PUBMED search), in their study of the disposition of ^14^C-ursodiol, a compound used to treat cholestasis without supportive data from pediatric populations [18,19]. This endogenous bile acid, like many pediatric drugs, required a tracer to differentiate between the dose and endogenous bile pools. The study was conducted to determine correct dosage of ursodiol in a group of low-weight infants under standard primary care at the Neonatal Intensive Care Unit of Loma Linda Children’s Hospital. This study was conducted with radioactive doses of 1–10 µCi, newborn weights of 1.2-2.0 Kg, blood sample volumes of 25 µl and 5–8 sampling points. The required blood loss was therefore less than 5% of the infant’s blood volume and can be obtained by heel-stick as well as from indwelling catheters. The co-administration of a large nonlabeled dose in Group B patients in this study did not change the pharmacokinetics of ursodiol for the radiolabeled microdose. Limited kinetic results from three neonates, each dosed at 1, 3.3 and 10 nCi of ^14^C-ursodiol with 48 h between doses, are provided in Figure 4. Overall, the results of this study demonstrated that AMS tracing is an effective, safe and noninvasive approach to studying drugs and nutrients in pediatric subjects, and in the extreme case in neonates no older than a few days.

Fortunately, the activity in pediatrics is only beginning and efforts are underway in Europe and possibly other regions to apply AMS. A European consortium is now undertaking long-term studies testing microdosing in pediatric populations [60,61]. Initial studies are designed to validate the approach using well-characterized adult drugs that are used in an off-label manner by clinicians. In particular the group not only demonstrated the feasibility of using a ^14^C-labeled microdose to study acetaminophen pharmacokinetics in young children, but also generated valuable metabolite disposition information. These studies are breaking new ground in high-density pharmacokinetic and metabolism readouts in infants and children. More studies can be expected as the AMS tools become more accepted within the community of pediatric investigators.

## New applications: biotherapeutics

Biotherapeutics (biologics, protein-based drugs, etc.) are a large class of molecules that include large molecular-weight proteins that have been designed to specifically mimic or inhibit endogenous human proteins, engineered antibodies using hybrid cell systems, and selective transporters of cytotoxic agents via antibody–drug conjugates (ADCs). AMS can bring the techniques of drug metabolism science to the challenging area of biotherapeutics.

The heterogeneity and dynamic nature of proteins and ADCs raises unique bioanalytical challenges that are not fully addressed by molecular mass spectrometry or ligand-binding assays. Radiolabeled biotherapeutics with AMS can be useful in bridging the gaps present between current analytical methods. AMS is a single method that delivers linear quantitation with universal lower limits of detection or quantification. We will briefly outline how the LLOQ is determined, with the understanding that this approach to LLOQ is universally applicable to any labeled molecule.

Biotherapeutics are heterogeneous in mass distributions and molar units are the logical unit of report. AMS is a molar instrument (mol ^14^C/mol ^12^C) whose ultimate sensitivity for quantifying isotopic labels in bulk tissues or fluids depends on four factors: the rarity of the isotope (background), the precision of the measurement, the number of isotope labels in the traced compound and the carbon content of the matrix under investigation. There are good estimates for the mean carbon content of common matrices and if not available, this value is obtained with an elemental analyzer. An LLOQ as six-times the standard deviation in the matrix background has been explained by Vogel *et al.* [3,62–63]; this value represents a 0.09% likelihood that normal distributions of the background and the sample would overlap with equivalent uncertainties. For singly labeled biologics measured to 1% precision (a function of acquisition time as in LSC), an LLOQ of ∼300 amol/ml of human plasma can be confidently predicted without making any actual determinations. For a doubly labeled protein, a limit of 150 amol/ml of plasma is predicted and so forth. In our experience, actual measured values will be within several percent of this ‘*in silico*’ estimate.

The first reported pharmaceutical use of AMS applied to a biologic was to assess the pharmacokinetics of ^14^C-labeled recombinant protein in rat [64]. The current pharmaceutical examples we discuss are: quantifying the maternal to fetal transfer of several biotherapeutics in multiple animal models, and a human microdosing kinetic study of a recombinant protein.

Understanding the exposure of a developing fetus to biotherapeutics is central to assessing potential risk. While large-molecular weight proteins are thought to have little chance of crossing the placenta, the placental permeability toward intermediate- and small-molecular weight proteins (∼10,000–50,000 Da) and newer classes of engineered proteins is largely uncharacterized. There is currently little consensus on what degree of transfer poses a risk to the developing fetus, so techniques that can detect transfer ratios (fetal blood/maternal blood level of 1%) would be useful. One industry group has undertaken a comparative bioanalytical study to assess the embryonic exposure of three tool molecules using three analytical platforms: AMS, LC/MS/MS and LSC [65]. The following is a summary of the results currently available from publication and presentations at scientific conferences.

Radiolabeled PEGylated adnectin [66], a domain antibody (dAb; a 40 KDa branched PEG attached to an 11 KDa adnectin protein) and a monoclonal antibody (mAb) were separately administered by iv. bolus doses (0.5–1 μCi) to pregnant guinea pigs (all three molecules) and cynomolgus monkey (mAb only). The dosing was timed to occur at the end of organogenesis, with administered radioactive doses sufficiently high to ensure detection in some maternal samples by LSC. Maternal blood samples were collected for 24 h over multiple time points postdosing with embryonic cord blood and whole fetuses harvested at 24 h postdosing. Neither LSC, nor LC/MS, displayed sufficient sensitivity to provide reportable results on placental transfer. AMS however, reported on transfer ratios down to 0.2% for PEG–Adnectin and PET–dAb in guinea pig with fg/ml sensitivity toward 10 μl sample collections. Placental permeability to mAb was significant in both species: transfer ratios of 1.5 and 3.5% were recorded for guinea pig and cynomolgus monkey, respectively. In the case of the mAb, these levels of transfer were seen as likely to have pharmacological effects on the developing fetus during organogenesis. This study demonstrates well how AMS can deliver critical data for decision-making using small samples and with minimal assay development.

Animal models are especially unsuitable for predicting high potentials for adverse events arising from immunogenicity. As a result, starting doses for human trials should be as low as reasonably possible without compromising the quality of the data [36]. Although the possibility for human testing was proposed under the FDA exploratory IND guidance document of 2006, with a protein-based drug microdose defined as ≤30 nmol, we are not aware of any early activities until a 2015 publication by the TNO group of the Netherlands [67]. The study showed, for the first time, that pharmacokinetic data of new biologic entities in humans can be successfully obtained early in the drug development process by the use of microdosing in a small group of healthy subjects combined with AMS. After only minimal preclinical testing, a first-in-human Phase 0/Phase I trial was performed with a human recombinant therapeutic protein (placental alkaline phosphatase [hRESCAP]) to assess its safety and kinetics. Pharmacokinetic analysis showed dose linearity from microdose (53 μg) [^14^C]-hRESCAP to therapeutic doses (up to 5.3 mg) of the protein in healthy volunteers. According to the authors the low-dosing regimens are inherently safe and only required small-scale GMP production facilities.

We anticipate more human pharmacokinetics investigations on large molecules now that the path has been prepared. We are also aware of other activities that have not yet been made publically available, such as the optimization of *in vivo* pharmacokinetics though bioconjugation alternatives (e.g., the type and extent of pegylation, choice of linker chemistry, etc.) and an application to quantify the uptake of active small molecules in tissues from antibody drug conjugates (ADCs). The latter application will prove to be valuable as this modality rises in importance.

## New technology: laboratory-sized AMS systems

Early AMS instruments were institutional-sized and operated at mega-eV energies, enlisting large Van De Graff acceleration tanks. While the instrumentation has dramatically reduced in size and operational complexity, many of the advances are evolutionary in impact. It may surprise some readers to learn that more than 20 sites now operate sub-MeV systems and another 100 AMS facilities have been set up world-wide [28]. Only a few of these facilities are offering services for biomedical sciences.

“*The ultimate goal of having available AMS instruments and the associated sample processing as easy to implement as conventional mass spectrometers has not yet been reached*.” [68].

This section focuses on developments in the AMS platform that are rendering it better-suited for meeting the demanding work flows of pharmaceutical science. These developments include: smaller laboratory-sized instruments, automated sample introduction via gaseous CO_2_ and liquid chromatographic interfacing to a gas-accepting ion source. It may be useful for the reader to be aware of the commercial options available for purchasing an AMS instrument. There are to the best of our knowledge three commercial options: National Electrostatics (NEC; USA), High Voltage Europa (HEEV; The Netherlands) and the Swiss-based IonPlus (which is a commercial spinout of the Laboratory for Ion Beam Physics at ETH Zürich, Switzerland). All three providers now offer their version of ‘compact’ instruments, dedicated to radiocarbon analysis, and all instruments have an option for a gas-accepting ion sources.

The evolution of AMS toward an emphasis on compact systems of decreasing operational complexity has been recently reviewed [68]. While the fundamental sector components of AMS spectrometry have changed little in 35 plus years, the development of charge-exchange collision cells using multiple collisions in dilute gas have reduced the energy required for destruction of molecular isobars (background), proportionately leading to a reduction in the size and operational complexity of instruments. Of significant note on the low-energy end of the spectrum is the compact AMS system namely MICADAS^TM^ developed at the Swiss Federal Institute of Technology [6,69] (ETH; Zürich, Switzerland; now sold by IonPlus) and the Single Stage open stack AMS system (SSAMS) manufactured by National Electrostatics Corp (NEC) (Middletown, WI, USA). The smallest systems (HEEV) are currently operating at or around 1 MeV and have multiple isotope analysis capabilities. As a result, they do not fall, in our view, into the same ‘compact’ or perhaps ‘ultracompact’ category as the other two instruments.

The MICADAS operates at a terminal voltage of 200 keV. These voltages are achieved using solid-state power sources in place of a traditional accelerator which enables the large reduction in overall size of the instrument. The instrument is vacuum-insulated which removes any need for the high-voltage protection cages found in other instruments. With a footprint of 2.5 m × 3 m and few special environmental controls, it establishes a benchmark for compact systems that can be installed in an average laboratory setting. Equally well-suited for biomedical purposes from a size, cost and maintenance perspective is the ‘compact’ SSAMS from NEC [70]. The design of the instrument is unique as carbon ions are accelerated via 250 keV open air deck and not by tandem acceleration (hence single-stage). It thus does not use an accelerator in the traditional sense, which allows for the reduction in size and operational complexity (Figure 5).

The physical limits in terms of overall size reduction have yet to be reached for AMS or AMS-like systems. For example, development projects at ETH Zürich include a 45 keV MICADAS system without any additional ion acceleration past the ion source; this system, if it becomes available, will represent mass spectrometric radiocarbon detection that no longer fully fits into the AMS designation. A more ‘eco-friendly’ version of the MICADAS, the GreenMICADAS, replaces power-consuming electromagnets with fixed magnets that results in a lower cost of operation (Figure 5).

The take-home message of this section is that the field is far from stagnant and one can envision in the coming years that systems comparable in complexity to standard isotope ratio mass spectrometry will become available. With concurrent progress in gas-accepting ion sources and automation to simplify sample handling, the instrument design should become less of a topic than the experimental data AMS empowers.

## New technology: direct injection CO_2_ interfacing & sample handling automation

AMS, due to its complexity and cumbersome sample preparation (graphitization) requirements, is still a niche, specialty technology for the pharmaceutical industry. As we are seeing continual improvements in instrument design, there are concurrent advances in simplification in how a sample can be introduced into the AMS ion source for measurement.

Graphitization or graphite production is the most common form in which a biological sample is introduced into AMS systems. To convert a biological sample to graphite is a two-stage process of oxidative combustion of the organic material to CO_2_, followed by reduction of CO_2_ to a fullerene (graphite) over an iron-group catalyst; the industry standard process as developed by Vogel [71] and improved by Ognibene [72] allows an operator to convert over 100 samples in a shift using septa-sealed reductive reaction vials. Even so, the procedure does not support the level of automation often needed to address the high workflows of the pharmaceutical industry. For example, a single metabolite radioprofile using fraction collection at higher temporal resolutions (e.g., 10-s fractions) can quickly generate a hundred or more samples. Thus, a typical AMS laboratory with a single instrument can process approximately five to six full radioprofiles per week depending on the size of each profile. When presented in these terms, the need for advances in throughput is apparent.

Elimination of the graphitization step through direct analysis of the organic CO_2_ combustion product is currently growing in use as operations refit their existing AMS instruments or purchase ready-made units. The direct injection of CO_2_ into an ion source was first demonstrated in the 1980s [27], but only in the last decade have gas accepting ion sources emerged as viable alternatives to graphitization. Advantages of introducing the sample as CO_2_ include a significant time saving in sample preparation, the capability to analyze submicrogram-sized samples (liquid chromatography fractions, single cellular analysis); and greater overall automation of the sample introduction process through direct couplings of separation and combustion systems such as gas chromatographs and elemental analyzers [73]. The notable drawbacks to gas analysis are lower ion beam currents and thus longer analysis times, some decrease in measurement precision and some risk of injection-to-injection carryover. The latter concern is particularly true for bio-AMS where sequential samples can vary by orders of magnitude in ^14^C contents.

Elimination of the graphite reduction step enables automated sample handling. We know of several facilities that have implemented various levels of automation through direct analysis of CO_2_, but, for the purposes of this paper, the activities of the AMS group at TNO in the Netherlands well exemplify the state of the art for automation as applied to a bio-AMS oratory [74].

The automated CO_2_ analyzing system at TNO can be simply divided into three subsections: automated combustion, sample trapping and staging and the gas-accepting ion source of the AMS instrument (1 MV high-voltage engineering). This setup allows direct analysis of 70 samples daily for a total ^14^C by AMS with no sample processing other than the pipetting of the sample into a tin foil cup for up to 200 AMS analyses before additional manual intervention. A simple schematic interface of the system is provided in Figure 6. Importantly, the results of validation-styled experiments are presented for a test compound ^14^C-acetominophen prepared in human plasma. Small aliquots of well-mixed plasma supplemented with ^14^C-acetominophen were analyzed for total radioactivity using the automated gas system. The results demonstrated that the automated technique showed a linear response over a 1200-fold range of 0.65–821 mBq/ml (0.039–51.9 dpm/ml). The LLOQ hovers at a value ∼10% higher than the natural background for ^14^C and corresponds to 0.67 amol (10^-18^) of acetaminophen. In terms of bioanalytical performance criteria, the system meets or exceeds current expectations for biological AMS validation criteria as set out by the European Bioanalysis Forum [41]. It is important to recognize, however, that the validation experiments were only performed as total ^14^C and not after liquid-chromatographic isolation of specific analytes. Compound isolation is the major source of error in the total measurement process and often the most time-intensive step. The study authors note that the next step will be to test the performance using liquid chromatographic isolates. In a personal communication with one of the authors, Dr W Vaes, it was confirmed that the system performs well with chromatographic isolates (after carbon supplementation) and these data are to be released soon.

The automated system is attractive from a time/cost perspective assuming robust, stable and continuous operation. The point of automation is to increase throughput while lowering labor and consumable costs. The current configuration addresses the latter, and we expect throughput will be driven higher through continued optimization in the operation of the elemental analyzers (duty cycle) and ion source efficiency. It will be interesting to see if automated systems lead to a net reduction in sample analysis pricing as provided by commercial CROs.

## New technology: true LC/AMS system

Direct coupling of an LC system to AMS has been a high development priority for many years. LC interfacing led to tremendous growth in the field of biological mass spectrometry and the same might be expected for AMS. The cesium sputter ion source used in virtually all AMS units cannot accept liquids so gaseous CO_2_ is the only practical option for mating LC with AMS. Until recently, the performance of gas-accepting ion source was inadequate to provide routine measurement support. The situation has changed with viable gas interfacing available on all the commercially available ion sources.

An early LC interface system was described at the Tannenbaum Laboratory at Massachusetts Institute of Technology (MIT) [75–77]. National Electrostatics Corp (NEC), MIT and GlaxoSmithKline subsequently entered into collaboration for an interface for direct analysis of CO_2_ produced by the laser combustion of liquid chromatograph eluate deposited on a copper oxide (CuO) substrate. The Automated Laser Gas Interface was reported to allow the measurement of 96 samples presented in the microtiter plate format in as little as 16 h. As attractive as this system appears, the precision and sensitivity of the early prototypes were relatively poor and there are no clear assurances that these shortcomings have been fully overcome at the time of writing this paper.

The group at Lawrence Livermore National Laboratory led by Ognibene and coworkers has developed a real-time moving wire interface (MWI) that appears highly promising [73,78–79]. The MWI was originally developed as an effluent monitor for liquid chromatography in the 1960s and later optimized and applied for liquid chromatograph/combustion isotope ratio mass spectrometry. The system consists of an HPLC operating at a flow rate of up to 125 μl/min that deposits the eluate as ‘drops’ onto an indented wire surface drawn at a feeding rate of 6 cm/s (see Figure 7). Higher flow rates are accommodated by the inclusion of a flow splitter, with the excess going to a fraction collector, a coupled mass spectrometric system for identification or a waste container. The wire travels into a drying oven for volatile solvent removal, then onward to a combustion oven set to 750°C under the flow of a carrier/oxidation gas mixture (95/5 helium:oxygen) feeding into a gas-accepting cesium sputter ion source (modified NEC source). Samples of less than 1 µg are analyzed with a detection limit of 50 zeptomol ^14^C/peak. The system has shown 100% efficiency in the transmission of carbon in nonvolatile analytes to a CO_2_-gas-accepting ion source, ensuring accurate results for metabolic profiling where differential loss of carbon mass would add variability to ‘peak’ area responses.

This MWI real-time direct coupling of HPLC to the AMS ion source will reduce the analysis time of complex metabolite separations from days to minutes. In principle, this development could be the ‘game changer,’ particularly for AMS as applied to metabolism studies that generate hundreds of AMS individual fractions using off-line fraction collection methods. It currently holds less potential for the quantitative analysis required for regulated pharmacokinetic studies in its current configuration. Different from the traditional fraction collection with dilution of carrier carbon to normalize the ^14^C to ^12^C beam, the normal mode of operation is as an ‘ultrasensitive ^14^C counter,’ not as an isotope ratio instrument *per se*; the normalization to stable carbon is confounded by biogenic carbon from the organic extracts injected onto the LC system. The strengths of the system lie in rapid and accurate metabolite screening profiling (all radiocarbon labeled compounds would provide equivalent responses) and high sensitivity toward very small samples whose radiocarbon signal would be diluted into the background after the addition of a carbon carrier with the traditional profiling approach. It is a major step forward in the evolution of AMS for biomedical research.

## New technology: radiocarbon laser-based detection systems

Although AMS has proven performance and continues to be improved upon, it is none the less a large instrument that is not trivial to install and maintain. Alternative detection techniques for ^14^C have been of great interest, with the vision of a simple laboratory-type instrument similar in operation to current forms of traditional mass spectrometry. Optical methods based upon laser spectroscopy in principle could achieve results competitive to AMS in a simpler and less expensive configuration.

The concept of using laser-based spectroscopic technologies for radiocarbon analysis has been around for some time; advances in coherent and tunable laser sources are facilitating progress toward useful benchtop optical radiocarbon detection systems. Currently described systems are in the prototype phase and have significant development work ahead before they may challenge AMS in terms of performance. None the less, these systems hold great promise and are worthy of discussion.

One device discussed extensively at radiocarbon conferences is the Intracavity Optogalvanic Spectroscopy (ICOGS) technique. ICOGS combines aspects of the laser-assisted ratio analyzer technique and intracavity absorption spectroscopy. High sensitivity is achieved via the laser optogalvanic effect, an electrical response to a gas discharge to an optical perturbation. The ICOGS system obtains AMS levels of sensitivity with a limit of detection down to 5 × 10^-^^15 14^C/^12^C (or parts-per-quadrillion) from submicromole CO_2_ samples, but lacks a linear response at ratios above 1.5 × 10^-^^12^, the region most useful for biological tracing. The ICOGS was believed, however, to have major scientific and commercial implications. As a result, at least five different research groups have attempted to replicate the system as originally proposed. The AMS group at Uppsala built a copy of the ICOGS system, but after several years of examination they were unable to reproduce the reported results on the signal dependence on ^14^C concentration and wavelength. It was concluded that the system over-estimated sensitivity by several orders of magnitude and that the nonlinearity of its ^14^C response would seriously limit its practical deployment [80]. Some researchers in the AMS community question whether the ICOGS technique is only measuring differences in ^14^C concentrations and early enthusiasm in now on hold pending new data.

A more recent development in laser-based detection systems is the cavity ring-down spectroscopy technique [81]. CRDS is a laser absorption technique where a laser pulse is stored in a high-finesse (reflective) optical cavity and allowed to bounce back and forth thousands of times though the absorbing species (gaseous CO_2_). The bouncing results in an effective light path lengths of up to a few kilometers, which increases sensitivity. The technique does not attempt to measure the absolute concentration of gas in the cell, but rather the rate constant (α) for the loss of the light within the cell, the so-called ‘ring down’ time. As a result, the technique is immune to variation in the light source intensity, which can be a significant source of error in conventional laser absorption methods.

A group at the Insituto Nazionale di Ottica-CNR (Florence, Italy) is developing the saturated-absorption cavity ringdown technique, a modification of the basic cavity ring-down spectroscopy technique for application specifically to biomedical and environmental carbon monitoring. A report comparing AMS, saturated-absorption cavity ringdown and liquid scintillation decay counting (LSC) described the performance parameters for these three methods using similar sets of samples [82]. The data show that the current laser prototype achieves the sensitivity of AMS for carbon dating (the most demanding measurement) in a format similar in size to a benchtop LSC, albeit with a lesser degree of precision (Figure 8). Two major hurdles to overcome, namely, multi-hour acquisition times and a need for relatively large volumes of CO_2_ (68 mg), remains to be addressed. The developers are confident that the fundamental concept is sound and that the obstacles can be surmounted from an engineering approach. A portable form of this system intended for atmospheric carbon monitoring is under commercial development in a partnership between the INO-CNR and Planetary Emissions Management.

The potential importance of this system is exemplified by parallel activity at the Lawrence Livermore National Laboratory, the original developers of biological tracing by AMS. A record search of the public NIH database finds that Lawrence Livermore National Laboratory was awarded 1R21GM111242 from NIH/NIGMS – for “Development of laser spectroscopic methods for quantification of ^14^C.” This proposal seeks to develop a table-top sized spectroscopic method using CRLS to quantify ^14^C in milligram-sized biological samples at levels down to the natural isotopic abundance.

The optical laser system(s) will also address a growing demand for monitoring the carbon economy and thus carry significant research and commercial implications for fields beyond biomedical research [82]. Many of the advances in interfacing technology to AMS can also be coupled to the optical laser system in principle, so advances in these supporting equipment have a future regardless of which radiocarbon detector system ultimately prevails.

## Conclusion

Radiocarbon studies coupled with sensitive detection systems provide insights into human metabolism in ways that can save cost by enabling ADME-type determinations without the regulatory overhead of higher levels of radioactivity. In this article we discussed the current state of AMS technologies most commonly applied to clinical drug development and highlighted a number of unique applications that are increasingly viewed as essential rather than merely beneficial options. We also discussed the current state of AMS technologies moving closer to frontline application in the pharmaceutical industry through the development of automated analysis systems and the direct coupling of AMS devices with liquid chromatographic systems. In the meantime, we are seeing the advent of a potentially very simple laser-based radiocarbon analysis system that could complement or even replace AMS as a core analytical technology. Such an event is not imminent in our view. If these technologies were simply a superior means of detecting radiocarbon, their value would lie in the reduction of radioactive doses and ability to work with minute samples. These are indeed benefits of importance, but the real power of AMS lies in the novel clinical study designs it enables. Such studies will offer greater value if they are performed sooner rather than later in clinical development.

## Future perspective

Revolutionary change is only possible with technology. Carbon dating tools from the world of ‘big physics’ now offer us new means to better understand the biochemistry of endogenous systems and the toxicology and pharmacology of xenobiotics and new agents being developed as pharmaceuticals. The adoption of a carbon dating instrument as an important tool in biomedicine is one of the more interesting examples of translational technological research. The benefits of new technology can often take years to realize, particularly in highly monitored industries such as pharmaceuticals. The opportunity to better understand new pharmaceuticals in diverse subpopulations of human without relying on stressful animal testing is now a permanent part of the new drug development landscape, regardless of which technology platform will dominate in the years ahead.

**Executive summary**

Accelerator mass spectrometry (AMS) is well established after 35 years and is used in bioanalysis to trace nutrients, toxins and therapeutics in humans.
Regulatory expectations for absolute bioavailability data have heightened interest in biomedical AMS.
AMS sensitivity allows ^14^C metabolic tracing in all subpopulations, including neonates and patients in impaired metabolic states.
The difficult analytical area of biotherapeutic absorption, distribution, metabolism and elimination will benefit from the specificity of radiolabel tagging leveraged with AMS sensitivity.
Compact AMS systems with automated sample handling and liquid chromatographic interfacing are becoming available.
Functional prototypes of optical laser systems are showing promise as a true benchtop radiocarbon detection system.
